# Breed-Specific Obstetric and Neonatal Parameters in Labrador Retrievers: Factors Influencing Early Neonatal Survival

**DOI:** 10.3390/ani16040600

**Published:** 2026-02-14

**Authors:** Piotr Andrzej Socha, Michela Beccaglia

**Affiliations:** 1 Department of Animal Reproduction with Clinic, Faculty of Veterinary Medicine, University of Warmia and Mazury in Olsztyn, Oczapowskiego Str. 14, 10-719 Olsztyn, Poland; 2 Reproforpets—Clinica Veterinaria Beccaglia, Via Napoli 5, 20841 Carate Brianza, MB, Italy; info@reproforpets.com

**Keywords:** canine reproduction, whelping, neonatal survival, stillbirth, maternal body weight

## Abstract

This study evaluated reproductive parameters, parturition characteristics, and early neonatal survival in 42 pregnant Labrador Retriever bitches and their 346 puppies between June and December 2024. The mean age of the bitches was 4.06 ± 1.59 years, and their average body weight was 33.14 ± 3.72 kg. Litter size averaged 8.24 ± 1.88 puppies, with an almost equal sex ratio (50.2% males; 49.8% females). Mean labour duration was 743 min, and intervals between successive births shortened initially before increasing in late parturition. In 57.1% of litters, all puppies were born alive. Early neonatal mortality was low: 0.6% within the first hour and 8.4% by day seven postpartum. A significant association was detected between prolonged parturition and a higher percentage of stillborn puppies (*p* = 0.001). Maternal body weight showed a substantial negative correlation with the number of live-born puppies (*r* = −0.554) and a positive correlation with stillbirths (*r* = 0.499). The exploratory Q coefficient, expressing the ratio between puppy and maternal body weight, differed significantly across survival categories (*p* = 0.0027), with surviving puppies displaying the highest ratio. Birth order significantly influenced mortality (*p* = 0.008), with a marked increase from the sixth puppy onwards. No effect of bitch age was observed on parturition length or puppy birth weight (*p* > 0.05). Overall, these findings suggest that maternal body weight, parturition duration, and birth order are important determinants of neonatal outcomes in Labrador Retrievers and may assist in improving perinatal management practices.

## Simple Summary

Labrador Retrievers are among the most popular dog breeds worldwide, yet comprehensive data on their normal whelping patterns and neonatal outcomes remain limited. This study analysed 42 bitches and their 346 puppies to identify factors that influence the course of parturition and early survival. Prolonged labour was strongly associated with a higher proportion of stillborn puppies, although overall neonatal survival remained high, with 91.6% of puppies alive at seven days postpartum. Higher maternal body weight was associated with fewer live-born puppies and increased neonatal mortality, and puppies with a lower body-weight ratio relative to their dam were more likely to die shortly after birth. Birth order also played an important role, with mortality increasing notably from the sixth puppy onward. These findings provide breed-specific reference values that may help clinicians and breeders optimise monitoring and neonatal care in Labrador Retrievers.

## 1. Introduction

The provision of obstetric and neonatal assistance in dogs should be grounded in comprehensive data regarding the physiological progression of parturition and newborn data. It is known that the perinatal period exerts a profound influence on the viability and welfare of the bitch and her offspring. Multiple maternal, foetal, and environmental factors influence outcomes during and following whelping, emphasising the necessity for breeders and veterinarians to meticulously monitor and manage the process [1,2]. While labour and neonatal parameters have been well defined in many domestic species, data for dogs remain limited [1,2,3].

In the event of complications arising during whelping, timely and accurate veterinary intervention is imperative to improve neonatal survival [2,3]. The reported puppy mortality rates vary between 8% and 20%, depending on breed, maternal condition, and perinatal management [4,5,6,7,8]. The temporal aspect of medical or surgical interventions has been demonstrated to exert a pivotal influence on neonatal outcomes [6]. Therefore, it is important to develop a comprehensive understanding of the predisposing factors associated with perinatal complications if we are to improve predictions of risk and facilitate the prevention of losses.

The breed of dog has been shown to have a significant effect on reproductive outcomes. As demonstrated in research, maternal body size, age, and parity are among the key determinants influencing the course of parturition [9,10]. Canine dystocia, otherwise known as difficult birth, is a prevalent obstetric complication associated with significant maternal mortality and stillbirth rates. Prolonged labour has been demonstrated to be directly correlated with an increased rate of stillbirths, with the highest losses observed after six hours of active whelping [3]. Incidence rates of dystocia have been reported to be approximately 5% of all parturitions [11]. However, the rate is highly variable within populations (3.7–28%) [4,12]. Furthermore, research has indicated that small and miniature breeds are more predisposed to dystocia [6]. Cornelius et al. [9] reported that the risk of dystocia increased with maternal age. Furthermore, dams with small litters (*n* < 5) and large litters (*n* > 9) were more likely to experience difficult whelping compared with those giving birth to medium-sized litters (*n* = 6). The risk of stillbirth is increased for large litters (>11 puppies), puppies with longer interbirth intervals, and is positively correlated with the duration of expulsion [6]. A more profound comprehension of the factors that contribute to dystocia can, as a consequence, enhance the efficacy of decision-making processes during the management of whelping, thereby leading to a reduction in mortality rates.

A number of studies have examined the association between specific breeds and the risk of stillbirth. Surveys have included Boxers [13], cross-breeds of Irish Wolfhounds, Leonbergers, Labrador Retrievers, and Newfoundlands [14], as well as groups comprising multiple breeds [15]. In the sole prospective study encompassing a whole cohort of puppies, lower body weight was identified as a risk factor for both stillbirths [14] and prolonged parturition [6]. Low and very-low birth weight have been repeatedly shown to markedly increase neonatal mortality across breeds [16]. Recent evidence suggests that proportionality indices such as the neonatal–maternal weight ratio can serve as effective predictors of neonatal viability [17].

Stillbirths and dystocia represent significant welfare and economic concerns for breeders and veterinarians. They are considered among the most devastating outcomes of canine pregnancy, yet treatment and preventive options remain limited. It is therefore vital for clinicians and breeders to comprehend the risk factors associated with stillbirths and dystocia, in order to facilitate informed decision-making when managing canine parturition in specific breeds.

The Labrador Retriever is a prominent large-breed dog that serves as a valuable model for the study of breed-specific reproductive parameters. The establishment of reference values for normal whelping characteristics has the potential to contribute to enhanced monitoring, early detection of abnormalities, and improved neonatal outcomes. Therefore, the aim of the study was to ascertain the obstetric and neonatal parameters that delineate normal and complicated births in the Labrador Retriever population. A comprehensive evaluation was conducted to ascertain the impact of various factors on the viability of the puppies. The primary parameters of interest were the litter size and gender ratio, inter-puppy intervals, the percentage of stillbirths, and birth weight of the puppies compared to the mother’s body weight observed prior to pregnancy in the anestrus phase of the estrous cycle. A dedicated questionnaire was utilised as a tool to collect data for this study. This research will provide practical reference data to support better breeding management and neonatal welfare in Labrador Retrievers.

The novelty of this work is to follow current trends in dog medicine including reproduction and obstetric examination trends concerning specific weight groups or breeds. Furthermore, purebred dogs are becoming increasingly valuable, and owners expect detailed, fact-based data on the course of a promising birth for puppies in order to limit losses. The results obtained using the Q coefficient developed for this study are very promising. This finding has the potential to make a substantial contribution to the field of canine reproduction by facilitating more accurate predictions of puppy survival rates. Such studies are particularly anticipated by clinicians and have a significant impact on the improvement of the performance of veterinarians in their daily work.

## 2. Materials and Methods

### 2.1. Animals

The present study was conducted between June 2024 and December 2024 and involved a total of 42 clinically healthy pregnant privately owned Labrador Retriever bitches and their respective litters. All examined bitches were housed in indoor–outdoor runs, fed a high-quality dry diet twice a day and provided with water ad libitum. All bitches were vaccinated and underwent diagnostic testing for the principal infectious diseases associated with canine abortion, including *Brucella canis*, *Canine herpesvirus* type 1 (CHV-1), *Leptospira* spp., and *Neospora caninum*, in accordance with current diagnostic standards.

Analysed parturitions were natural and uncomplicatedly occurring, with all procedures carried out as part of routine veterinary care. Births occurred in specially adapted breeding kennel facilities or in veterinary clinics. All deliveries were under the direct supervision and support of one of two experienced veterinarians, the authors of this article and the breeders who owned the bitches included in this study.

The mean age of the bitches was 4.06 years (SD = 1.59), ranging from 1.5 to 8 years. The age distribution of 42 Labrador Retriever bitches participating in the study was as follows: the ‘young’ group (<3 years) included 9 bitches (21.43% of the total), the ‘middle-aged’ group (3–5 years) included 19 bitches (45.24% of the total), and the ‘older’ group (≥5 years) included 14 bitches (33.33% of the total).

The mean body weight was 33.14 kg (SD = 3.72), with a range from 27 kg to 39 kg. A thorough analysis of the reproductive history revealed that the mean number of previous pregnancies was 2.10 (SD = 0.98). This finding indicates that the majority of the bitches had experienced at least one prior gestation. The minimum and maximum values recorded for this category were one and four pregnancies, respectively. The mean gestation length calculated from the first day of mating was found to be 60.17 days (SD = 5.14), with a range from 57 to 64 days. The characteristics of the 42 Labrador Retriever bitches that were examined are presented in Table 1.

### 2.2. Questionnaire

The data were collected using a structured questionnaire, which was completed for observed parturitions in Labrador Retrievers. All evaluated deliveries were natural and conducted under the close supervision of a veterinary professional. During each whelping, specialised veterinary assistance was mandatory in order to ensure appropriate monitoring of the process and to protect the health and welfare of both the dam and her offspring.

Veterinarians supervised the whelping process but did not provide medical or surgical treatment in any included case. Cases in which it was necessary to implement pharmacological or surgical interventions, including oxytocin administration, fluid therapy, and caesarean section (C-section), were excluded from the study in order to ensure uniformity in the dataset.

Completion of the questionnaires was undertaken by veterinarian or by the owners (with a full verification of the information) until the arrival of the veterinarian, who was provided with detailed instructions to ensure accurate and consistent reporting. Following a rigorous examination, it was determined that both owner compliance and the reliability of the collected data met the requisite standards for inclusion in the subsequent analysis.

### 2.3. Data Collection

The following parameters were analysed: the age (in years) and body weight (in kilograms) of the bitch, the length of gestation (in days), the total duration of expulsion (in minutes), the inter-puppy interval (in minutes), the litter size, the birth weight (in grams) of the puppies, and the number of stillborn puppies. In addition, for the purposes of the study, a model coefficient was created, which was designated the Q coefficient.

The length of gestation was calculated as the number of days between the date of the first confirmed mating and the birth of the first puppy. The inter-puppy interval was defined as the time elapsed between the expulsion of two consecutive puppies, and also as the time elapsed between the expulsion of subsequent puppies relative to the first puppy. The litter size was recorded as the total number of puppies born, irrespective of viability. The measurement of birth weight was undertaken immediately following expulsion. This was achieved by means of a calibrated electronic scale. The sex of each puppy was determined and recorded at birth. Stillborn puppies were defined as those showing no signs of life at birth and not responding to resuscitation attempts within 15 min.

### 2.4. Statistical Analysis

Statistical analyses were performed using Statistica Software, version 13 (StatSoft, Kraków, Poland), which enabled comprehensive data processing and interpretation. The graphical presentation of the results was performed using GraphPad Software, version 10 (GraphPad, San Diego, CA, USA).

In order to provide an accurate characterisation of the studied population of Labrador Retrievers and to assess significant relationships between variables, a range of statistical methods was applied. These included descriptive statistics, correlation analysis, and analysis of variance (ANOVA). The statistical significance was set at *p* < 0.05.

Prior to analysis, the assumptions of linearity and normality were verified. Linearity was assessed using two-dimensional scatter plots of the analysed variables, while the assumption of normal distribution was tested using the Shapiro–Wilk test.

Quantitative variables were expressed in terms of arithmetic mean (M), standard deviation (SD), median (Me), and quartile values (Q1—lower quartile; Q3—upper quartile). This permitted an assessment of both central tendency and data dispersion. Furthermore, the skewness and kurtosis values were calculated in order to evaluate the shape of the variable distributions. The mode (Mo), representing the most frequently occurring value, was also included in the analysis.

The variability of the expulsion time between successive puppies was assessed using the coefficient of variation (V). The degree of variability was interpreted in accordance with the following classification system. The variability is low or minimal when V is less than 25%. The variability is considered to be average when the percentage falls between 25% and 45%. The variability is categorised as high when the percentage falls between 45% and 100%. The variability is categorised as ‘very high’ when V is equal to or greater than 100%.

The relationship between quantitative variables was determined using Pearson’s linear correlation coefficient (*r*). The following interpretation was made of the strength of the correlation. The data demonstrate a weak correlation between 0.00 and 0.30. The correlation coefficient ranges from 0.30 to 0.50, indicating a moderate correlation. The data indicate a strong correlation between 0.50 and 0.70. The data indicate a very strong correlation between the variables at 0.70–1.00. The presence of positive coefficients indicated an increase in both variables concurrently, whilst negative coefficients suggested an inverse relationship between the variables. The presence of coefficient values approaching zero indicated an absence of any significant correlation between the parameters under analysis.

In order to compare the mean values of the variables across the different groups, a one-way ANOVA was applied. The homogeneity of variance was verified using Levene’s test. In instances where statistically significant differences were identified, Tukey’s HSD post hoc test was employed to ascertain which groups exhibited substantial differences. For each analysis of variance (ANOVA) model, the F-statistic and the corresponding *p*-value were reported in order to facilitate the evaluation of the model.

In consideration of the research findings, the coefficient was formulated and designated the Q coefficient. This coefficient appeared associated with survival rate in this dataset. The value of the Q coefficient is calculated by dividing the puppy body weight by the bitch body weight in the anestrus period and then multiplying the result by 100. The Q coefficient was explored as a potential proportionality index relating puppy to maternal body weight.

## 3. Results

### 3.1. Litter Size and Gender Ratio

**Figure 1:**
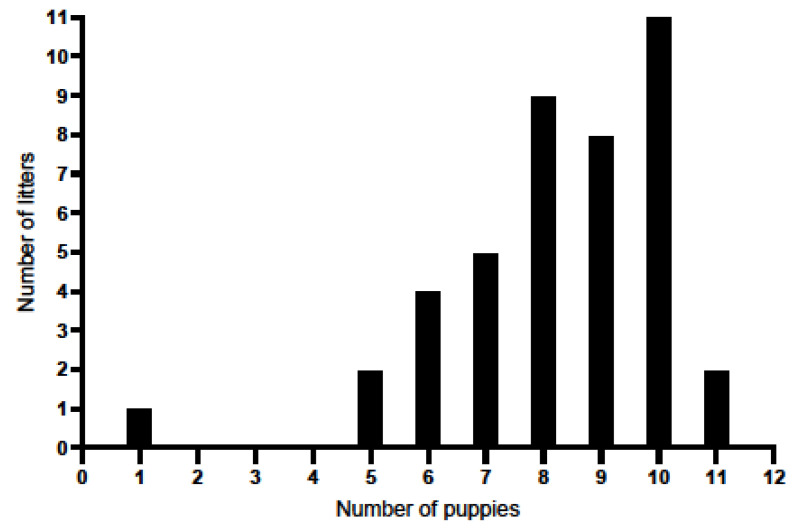
Litter size of the studied Labrador Retrievers.

The analysis included a total of 42 litters, with a total of 346 puppies being analysed. No statistically significant differences in litter size were found among the age groups of bitches (intercept: df = 1, F = 688.97, *p* < 0.001; for groups: df = 2, F = 1.35, *p* = 0.27). The mean litter size was 8.24 (SD = 1.88) puppies, with a median of 8.5 and a range of 1 to 11 (see Figure 1 and Table 2), indicating relatively consistent reproductive performance among the bitches under study.

**Figure 2:**
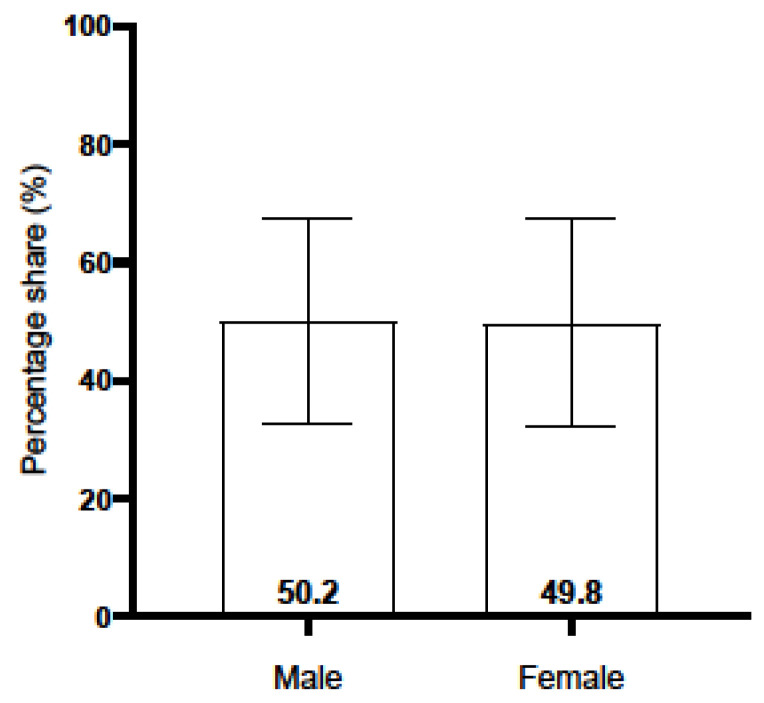
The gender ratio in Labrador Retriever litters.

With regard to gender distribution, the mean number of male puppies per litter was 4.14 (SD = 1.66), while the mean number of female puppies was 4.10 (SD = 1.64). The observed sex ratio at birth was found to be almost equal, with males accounting for 50.2% of the population and females for 49.8%. This finding is illustrated in Figure 2.

### 3.2. Inter-Puppy Intervals

The average total duration of labour in bitches was 743 min (approximately 12 h and 23 min). The initial point of departure for the analyses was the onset of labour. In the cases under consideration, the delivery time for the first puppy was found to be zero minutes for all bitches.

**Figure 3:**
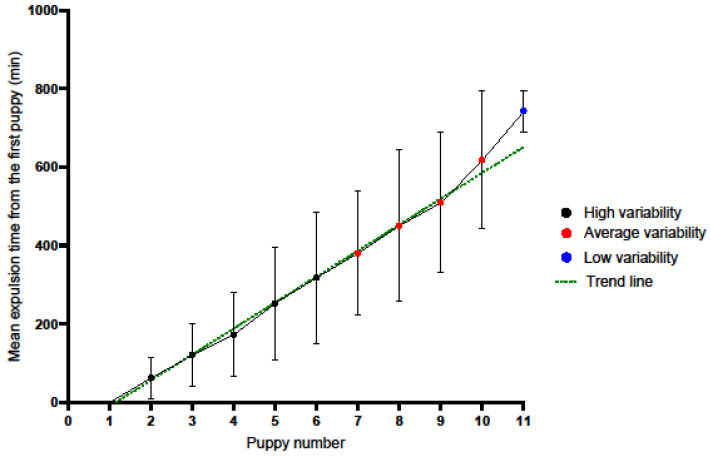
The average time between the birth of successive puppies (relative to the first puppy), together with the standard deviation and level of variability.

The results pertaining to the time elapsed between the birth of successive puppies in relation to the first puppy are presented in Figure 3 and Table 3.

Subsequent whelping times (counting from the first puppy) show considerable variation (see Table 3). The second puppy appeared on average after 62.51 min (SD = 53.65, n = 41, V = 85.82%, high variability), the fifth puppy appeared on average after 253.10 min (SD = 143.53, n = 41, V = 56.71%, high variability), the ninth puppy appeared on average after 509.62 min (SD = 179.29, n = 21, V = 35.18%, medium variability), and the eleventh pup appeared on average after 743.00 min (SD = 52.33, n = 2, V = 7.04%, low variability). Birth intervals tended to increase in later stages of labour, while variability progressively decreased.

**Figure 4:**
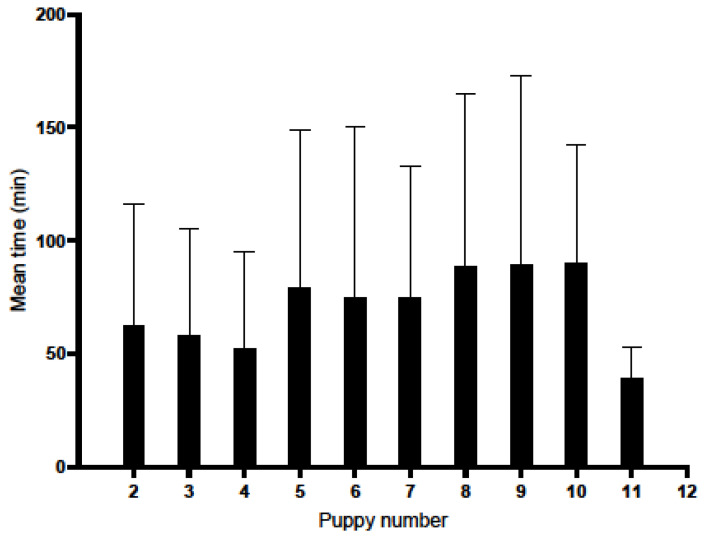
The time between the expulsion of two consecutive puppies during birth. The bar chart shows the average time of appearance of subsequent puppies from the first (reference point ‘0’) to the eleventh, together with standard deviations.

Figure 4 and Table 4 show the expulsion times of two successive puppies during labour. There is considerable variation in the time between births, particularly in the middle of the litter. The second puppy appeared on average after 62.51 min (SD = 53.65, n = 41, V = 85.82%, high variability), the fifth puppy appeared on average after 79.59 min (SD = 69.43, n = 41, V = 87.23%, high variability), the sixth appeared on average after 75.31 min (SD = 75.21, n = 39, V = 99.87%, high variability), the tenth appeared on average after 90.62 min (SD = 52.03, n = 13, V = 57.42%, average variability), the eleventh puppy appeared on average after 39.50 min (SD = 13.44, n = 2, V = 34.01%, average variability).

The analysis of the variability of the puppy expulsion time indicates that, in the initial phase of parturition, the intervals between subsequent puppies are reduced, whilst in the later stage, the time between births is increased. No statistically significant differences in the duration of parturition were found among the age groups of bitches (intercept: df = 1, F = 143.97, *p* < 0.001; for groups: df = 2, F = 0.24, *p* = 0.79).

### 3.3. Stillborn Puppies and Neonatal Survival

The mean number of stillborn puppies per litter was 0.67 (SD = 0.93), representing approximately 8.4% of all puppies born (see Table 5).

In 24 of the 42 litters (57.1%), all puppies were born alive. The number of stillborn puppies per litter ranged from 0 to 4.

Neonatal mortality within the first hour after birth was found to be minimal, with a mean value of 0.05 puppies per litter (SD = 0.22), representing approximately 0.6% of all live-born puppies. In 40 out of 42 litters, all neonates survived the first hour of life. No further fatalities were documented within the initial 24 h period postpartum.

On the seventh day after parturition, the mean number of surviving puppies per litter was 7.31 (SD = 1.99), corresponding to a neonatal survival rate of approximately 91.6%.

One-way ANOVA analysis showed a significant difference (t = 3.75; *p* = 0.001) in the short parturition group, in which the labour duration was shorter than the median value of the labour duration (mean percentage of stillborn puppies in this group was 6.1% with SD = 2.8) compared to the long parturition group (mean percentage of stillborn puppies was 12.1% with SD = 3.2). Longer parturition was significantly associated with increased stillbirth rates.

### 3.4. Birth Weight

**Figure 5:**
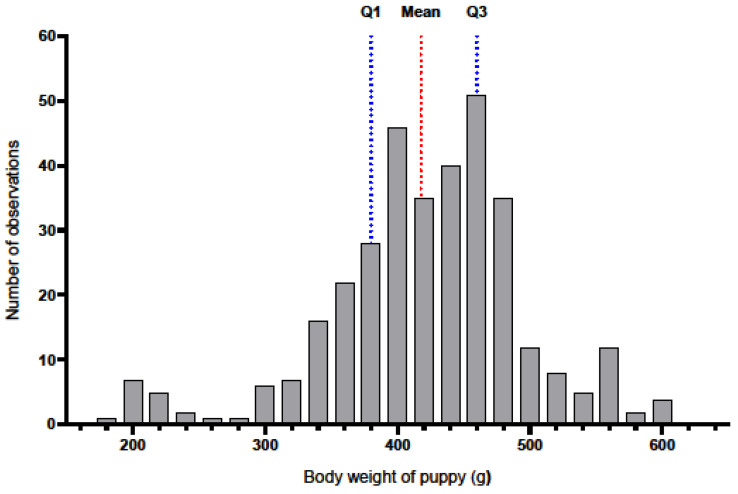
Histogram of puppy body weight based on the number of observations.

In a study of 346 Labrador Retriever puppies, the mean birth weight was found to be 417.60 g (SD = 75.65), as illustrated in Figure 5.

The mean body weight of the puppies was M = 417.60 g, SD = 75.65. The median was Me = 420.00 g, and the mode had the same value (Mo = 420.00 g), suggesting that the most common birth weight of puppies was 420 g. The lower quartile was Q1 = 380.00 g, and the upper quartile was Q3 = 460.00 g, meaning that half of the puppies had a body weight in the range of 380 g to 460 g. No statistically significant differences in puppy body weight were observed among the age groups of bitches (intercept: df = 1, F = 9605.97, *p* < 0.001; for groups: df = 2, F = 2.04, *p* = 0.13).

### 3.5. The Q Coefficient Model

The proposed coefficient, denoted Q, is a calculation that considers the birth weight of the puppy relative to the dam body weight, which was observed prior to pregnancy in the anestrus phase of the estrous cycle. The result was then multiplied by 100. The mean value of the Q coefficient was found to be M = 12.75, SD = 2.32 (see Table 6).

The median was Me = 12.58, and the mode was slightly higher at Mo = 13.33. The lower quartile was Q1 = 11.62, and the upper quartile Q3 = 13.75, indicating that 50% of the observations fell within this range. The skewness of the distribution was found to be 0.21, indicating a slight rightward skewness. The kurtosis was found to be 2.11, indicating a distribution that is more elongated, with a greater number of outlying values.

### 3.6. Correlation Between the Body Weight of Bitches Measured Prior to Pregnancy in the Anestrus Phase of the Estrous Cycle and Several Key Metrics, i.e., the Number of Live-Born Puppies, the Number of Stillborn Puppies and the Survival Rate of Puppies in the First Seven Days of Life

Pearson’s r test revealed a strong negative correlation between the body weight of the bitches, measured before pregnancy in the anestrus phase of the estrous cycle, and the number of live-born puppies (−0.554).

The correlation between the body weight of the bitches, measured before pregnancy, and the number of stillborn puppies (0.499) was moderately positive.

A strong negative correlation is evident in the data. In particular, the number of live puppies at seven days of age was found to have a significant negative correlation with the mean value.

### 3.7. Determination of the Q Coefficient and Puppy Survival Rate

The analysis was conducted using a one-way analysis ANOVA method. The statistical analysis yielded a highly significant result (F = 590.67; *p* < 0.001), indicating that the Q coefficient exhibited a substantial difference within the studied population. Puppy survival rate: This is a statistically significant factor (F = 4.82; *p* = 0.0027), indicating that there are substantial differences in the Q coefficient ratio between the various survival groups. The random error value (MS = 5.226) indicates moderate data variation.

Tukey’s HSD post hoc test is utilised for the purpose of determining any significant differences between survival groups. The lowest Q coefficient ratio was observed in puppies that died within an hour (7.97). Puppies that were stillborn or died within seven days exhibited a higher weight ratio (approximately 11.90–12.05). Surviving puppies had the highest Q coefficient values, suggesting a relationship between proportional birth weight and survival (12.88).

The results indicate that puppy survival was significantly associated with the Q coefficient ratio. Puppies with a lower Q coefficient were more likely to die within the first hours of life. Puppies that were stillborn or died within seven days exhibited Q coefficients that were similar to each other but still lower than those observed in surviving puppies. The analysis revealed that the puppies with the highest Q coefficient were the ones that survived. This finding suggests that a greater birth weight of the puppy relative to the bitch’s body weight is a factor that favours survival.

### 3.8. The Number of Puppies in a Litter and Their Survival Rate

The one-way ANOVA revealed a statistically significant impact of free expression (F = 787.21; *p* < 0.001), and it was used to compare litter size among survival outcome groups. Puppy survival rate: The analysis revealed that this factor was not statistically significant (F = 1.021; *p* = 0.383), indicating that there were no substantial differences in the number of puppies in a litter across different survival categories. The random error value (MS = 2.318) indicates moderate variation in the data.

Tukey’s HSD post hoc test (although no significant differences were found in ANOVA, Tukey’s test allows for the assessment of trends between groups) was utilised. The lowest number of puppies per litter was observed in the group where puppies died within seven days (7.89). The highest number of puppies was observed in the group where the puppies died within an hour (9.00). The average number of puppies per litter was found to be similar for both live and stillborn puppies, with an average of approximately 8.66–8.89.

The lack of statistically significant differences suggests that the number of puppies in a litter does not directly impact puppy survival. The observed trend suggests that larger litters may be associated with a slightly elevated risk of early puppy mortality.

## 4. Discussion

The present study is a unique and comprehensive one based on a group of 42 pregnant Labrador Retrievers and 346 puppies. It provides valuable information on the obstetric and neonatal parameters that exert a significant influence on reproductive outcomes in this particular breed. The results of the study demonstrate the key role of maternal body weight, measured prior to pregnancy, the duration and progression of parturition, and the relative birth weight of puppies in determining perinatal and early neonatal survival in Labrador Retrievers.

### 4.1. Litter Size

Many factors have been found as potentially influencing litter size, including breed, the size of the dog, the age of the bitch, season of the year, number of matings, mode of mating (natural or artificial insemination), and the quality of the semen [2,9,14,18,19,20]. It has been shown that in large breeds (25–45 kg) the mean litter size increased significantly with size of the breed [20].

In our study the mean litter size was 8.24 puppies and is comparable with previously reported results for other large dog breeds where the mean litter size ranged from 6.9 [20] to 7.6 [14]. What is more, it has been established that the larger the breed, the greater the number of puppies that can be born in a litter, with the potential for up to 11–12 puppies [2,18,19,20]. Our results are consistent with this information. The maximum number of puppies per litter in Labrador Retrievers was 11. The comparatively low degree of variability in litter size indicates reproductive homogeneity within the sampled population of Labrador Retrievers. However, it is important to note that among the studied population of 42 pregnant Labrador Retriever bitches, one pregnancy was a singleton. The delivery process was normal; thus, this result was also included in the study. In dogs of larger breeds, the small size of the litter is of particular significance, as a limited number of puppies may be incapable of producing sufficiently robust signals to induce labour [2,13]. The maternal age is believed to have an influence on litter size. In the present study, singleton pregnancy occurred in a bitch that was giving birth for the third time in the age of five years. In accordance with the findings of previous studies, bitches that have reached six years of age and older are predisposed to a higher risk of dystocia [2,19]. In this study, the mean age of bitches was 4.06 years, which corroborates with earlier reports indicating that female dogs aged between two and five years give birth to more puppies per litter [2,19].

Regarding the sex ratio at birth, it remained balanced and almost equal, with males accounting for 50.2% of the population and females for 49.8%, which is consistent with prior studies indicating that sex ratios in dog litters rarely differs significantly from parity [19,20].

Furthermore, the analysis revealed no significant differences in litter size across groups with varying neonatal survival outcomes. While the results indicated a tendency towards slightly larger litters in cases involving early neonatal mortality, these differences did not attain statistical significance. This finding lends further support to earlier conclusions that neonatal survival in dogs is influenced by a broad range of factors, including genetic, maternal, environmental, and peripartum factors, and cannot be attributed solely to litter size [6,9,19,20,21].

### 4.2. Parturition Dynamics and Their Impact on Neonatal Mortality

We found the duration of parturition as a critical factor associated with neonatal survival. The average duration of parturition in bitches was 743 min (approximately 12 h and 23 min). Bitches with prolonged expulsion phases, when the labour duration was longer than the median value of the parturition duration, had a significantly higher proportion of stillborn puppies compared with those undergoing shorter labour. This result is consistent with previous research demonstrating that extended parturition increases the risk of foetal hypoxia, dystocia, and intrapartum death [2,9]. The critical importance of timely intervention has also been demonstrated in studies evaluating decision-to-delivery intervals and foetal survival during dystocia [22].

Our results indicate that in the initial phase of parturition, the intervals between subsequent puppies were reduced compared to the later stages. The phenomenon of pronounced prolongation is frequently associated with uterine inertia or exhaustion [2,9,23,24,25]. The significant increase in neonatal mortality observed from the sixth newborn onward in the present study lends further credence to the hypothesis that later-born puppies are more susceptible to intrapartum compromise.

It should also be highlighted that in the present study, no effect of bitch age was demonstrated on pregnancy duration, litter size, puppy body weight, or duration of parturition. These findings are consistent with previous reports that maternal age within normal reproductive limits exerts a negligible effect on parturition efficiency and foetal growth [2,19].

### 4.3. Maternal Body Weight, the Q Coefficient, and Neonatal Survival

In the present study one of the most interesting findings was the strong association between maternal body weight measured prior to pregnancy during anestrus phase and perinatal outcomes. The negative correlation between the bitch body weight and the number of live-born puppies, alongside the positive correlation with stillbirths, suggests that higher maternal body weight was associated with poorer reproductive outcomes in this cohort. It is noteworthy that the average body weight of the examined Labrador Retriever bitches was 33.14 kg. However, the relationship between body fat mass and body condition score (BCS) was not investigated in this study. This is a significant area for future research in order to enhance the precision of the results. Moreover, obesity is a significant risk factor for parturition complications, exerting its influence on uterine contractility mechanisms. In obese women [26] as well as dogs [27,28,29], adipocyte infiltration into the uterine smooth muscle predisposes to contractile insufficiency. This may therefore be a cause of uterine inertia and, consequently, delayed parturition.

The Q coefficient was developed for the specific purpose of this study. The Q coefficient, representing the ratio of puppy body weight to maternal body weight measured prior to pregnancy during anestrus phase, and multiplied by 100, proved to be a valuable indicator of neonatal viability. The mean value was found to be 12.75. Puppies with a lower Q coefficient were more likely to die within the first hours of life. Puppies that were stillborn or died within seven days presented Q coefficients very similar to each other but still lower than those observed in surviving puppies. The analysis revealed that the puppies with the highest Q coefficient were the ones that survived. This finding suggests that a greater birth weight of the puppy relative to the bitch’s body weight is a factor that favours survival. Comparable findings have recently been reported for the neonatal–maternal weight ratio, reinforcing the clinical relevance of proportionality metrics [17]. In the present study, puppies that died within the first hour had the lowest Q values, while those surviving the first week had the highest. These results align with the broader understanding that insufficient intrauterine growth compromises neonatal thermoregulation, immune competence, and survival chances [2,30,31]. Further research is required to validate the Q coefficient not only within the Labrador Retriever breed, but in other breeds as well.

### 4.4. Neonatal Survival

Neonatal mortality rates in this study were low compared with previously published data, which commonly report early neonatal mortality ranging from 3% to 7% in large and medium breeds [17]. Furthermore, it was observed that more than half of the litters were not affected by stillbirth, and minimal puppies died within the first hour after birth (0.6%). The survival rate of 91.6% by day seven is consistent with the outcomes typically observed in litters provided with optimal veterinary management and environmental conditions [1].

The main determinants of neonatal loss identified in this study—prolonged parturition, increased maternal body weight, and a low Q coefficient—correspond well with established risk factors described in canine neonatology literature [2,7,21].

### 4.5. Limitations

It is imperative to acknowledge the limitations inherent in this approach. All parturitions took place under veterinary supervision, which may have reduced the incidence of complications compared with settings lacking continuous monitoring. Moreover, the study’s restriction to Labrador Retrievers enhances internal validity but limits generalisability across breeds. Comparative studies across breeds with varying conformational and physiological traits would further clarify the universality of the observed trends.

## 5. Conclusions

This study provides novel information on reproductive performance, parturition dynamics, and neonatal outcomes in Labrador Retriever bitches and their puppies. The findings of this study demonstrate that body weight of the bitch, the duration of parturition, and the proportionality between puppy body weight and body weight of the mother, measured prior to pregnancy in the anestrus phase (Q coefficient), are main determinants of neonatal and early neonatal survival. Prolonged labour was found to be associated with a significantly increased risk of stillbirth, particularly in puppies born later in the expulsion sequence. Furthermore, an increased maternal body weight was found to be associated with a reduced number of live-born puppies and elevated rates of neonatal loss. These findings emphasise the importance of pre-breeding and gestational weight management strategies to ensure optimal outcomes.

The Q coefficient was identified as a potentially useful indicator of neonatal viability, with higher values indicative of improved survival in the first week of life. In contrast, puppies with low birth weight exhibited a reduced chance of survival during the immediate postpartum period. The litter size did not have a significant impact on overall neonatal survival, and there was no effect of maternal age on either pregnancy duration, litter size, puppy birth weight or parturition duration.

The results of this study highlight the complex and multifactorial determinants of reproductive success in dogs. It is therefore essential to closely monitor maternal condition, labour progression, and neonatal proportionality. The implementation of these findings in breeding and clinical practice has the potential to contribute to a reduction in perinatal mortality and an enhancement in overall reproductive outcomes in Labrador Retrievers.

## Figures and Tables

**Table 1:** Characteristics of the Labrador Retriever bitches included in the study.

Parameter	Mean ± SD	Minimum	Maximum
Age (years)	4.06 ± 1.59	1.5	8
Body weight (kg)	33.14 ± 3.72	27.00	39.00
Number of previous pregnancies	2.10 ± 0.98	1	4
Gestation length counted from the first mating	60.17 ± 5.14	57	64
Number of bitches	42	-	-

SD—standard deviation.

**Table 2:** The total number of puppies in the litter.

Statistical Measure	Value
Mean (M) with standard deviation (SD)	8.24 ± 1.88
Median (Me)	8.5
Mode (Mo)	10
Lower quartile (Q1)	7
Upper quartile (Q3)	10
Skewness	−1.37
Kurtosis	3.24
Minimum	1
Maximum	11

**Table 3:** The inter-puppy intervals between the birth of successive puppies in the Labrador Retriever related to the birth of the first puppy (reference point ‘0’).

Puppy Number	Mean Expulsion Time from the First Puppy (min)	Standard Deviation (SD)	Number of Cases (n)	Coefficient of Variation V (%)	Assessment of Variability
2	62.51	53.65	41	85.82	high
3	120.90	78.51	41	69.94	high
4	173.51	107.94	41	62.21	high
5	253.10	143.53	41	56.71	high
6	318.44	167.64	39	52.65	high
7	381.06	157.51	35	41.33	average
8	451.30	192.55	30	42.67	average
9	509.62	179.29	21	35.18	average
10	618.15	175.56	13	28.40	average
11	743.00	52.33	2	7.04	low

**Table 4:** The inter-puppy intervals in the Labrador Retriever counted between subsequent puppies from the first puppy (reference point ‘0’).

Puppy Number	Mean Expulsion Time from the First Puppy (min)	Standard Deviation (SD)	Number of Cases (n)	Coefficient of Variation V (%)	Assessment of Variability
2	62.51	53.65	41	85.82	high
3	58.39	46.60	41	79.81	high
4	52.61	42.41	41	80.61	high
5	79.59	69.43	41	87.23	high
6	75.31	75.21	39	99.87	high
7	75.37	57.75	35	76.62	high
8	88.90	75.79	30	85.25	high
9	89.71	82.84	21	92.34	high
10	90.62	52.03	13	57.42	average
11	39.50	13.44	2	34.01	average

**Table 5:** Evaluation of mortality in the Labrador Retriever puppies.

Parameter Stage	Mean ± SD	Percentage (%)	Range	Additional Information
Number of stillborn puppies at birth	0.67 ± 0.93	8.4	0–4	In 24 cases of 42 all puppies were born live
Mortality within the first hour of life	0.05 ± 0.22	0.6	0–1	In 40 of 42 cases, all puppies survived the first hour. No addition deaths were recorded after 24 h
Number of live puppies at 7 days	7.31 ± 1.99	91.6	1–10	Mean litter size at birth: 8.24; median = 8.5 indicating that most litters maintained a number of puppies close to that at birth

**Table 6:** Table for descriptive statistics for the Q coefficient.

Statistical Measure	Value
Mean (M) with standard deviation (SD)	12.75 ± 2.32
Median (Me)	12.58
Mode (Mo)	13.33
Lower quartile (Q1)	11.62
Upper quartile (Q3)	13.75
Skewness	0.21
Kurtosis	2.11

## Data Availability

The data presented in this study are available on request from the corresponding author.
